# Progression and diagnostic challenges of desmoplastic infantile ganglioglioma in a non-infant: a case report with 5-year follow-up

**DOI:** 10.3389/fonc.2025.1411213

**Published:** 2025-02-10

**Authors:** Yan Yang, Xuzhu Chen, Xin Liu, Shiguang Li

**Affiliations:** ^1^ Department of Radiology, The Affiliated Jinyang Hospital of Guizhou Medical University, Guiyang, China; ^2^ Department of Radiology, Beijing Tiantan Hospital, Capital Medical University, Beijing, China; ^3^ Department of Pathology, Beijing Tiantan Hospital, Capital Medical University, Beijing, China

**Keywords:** desmoplastic infantile ganglioglioma, tumor progression, non-infant, magnetic resonance imaging, case report

## Abstract

Desmoplastic infantile ganglioglioma (DIG) is a rare intracranial benign tumor occurring in infants under 2 years of age. It has good biological and behavioral characteristics and occasionally has malignant characteristics, such as multiple intracranial lesions, postoperative progression or recurrence, meningeal diffusion, and metastasis. We present a non-infant with DIG who underwent tumor progression. A 16-year-old girl presented with DIG in the cerebral cistern and underwent subtotal resection. A magnetic resonance imaging (MRI) of the brain 2 years later revealed that the area of abnormal enhancement in the surgical site was approximately the same as before, and follow-up was continued. A reexamination 5 years later showed that the residual extent of the operative area was significantly larger than before and involved the right frontal and temporal lobes, considering the progression of the residual part of the tumor. This case report focuses on the occurrence of DIG and its potential malignant features, as assessed through magnetic resonance imaging.

## 1 Introduction

Desmoplastic infantile ganglioglioma (DIG) is a rare supratentorial tumor that accounts for 0.1%–1% of childhood central nervous system (CNS) tumors and is most common in infants under 2 years of age (1). DIG is classified as a desmoplastic CNS neoplasm and has been categorized as grade 1 by the World Health Organization (WHO) due to its benign tumor behavior (2). If surgical resection is complete, the patient has a good prognosis without radiotherapy and chemotherapy. However, the benign nature of this tumor has been questioned with successive reports of atypical, progressive, and multifocal tumors (1, 3). Here, we report a rare case of DIG in a non-infant who underwent tumor progression after subtotal resection.

## 2 Case presentation

A 16-year-old female patient experienced limb twitching for the first time 15 years ago (9 months after birth) without any apparent cause, which persisted for several seconds before spontaneous remission. She was taken to a local hospital, where she received no treatment, the details of which are unknown. Afterward, seizures occurred approximately once every 1 to 2 months. When she was two years old, she was sent to a local hospital for a computed tomography (CT) scan of her head, which found no abnormalities. She was taking antiepileptic drugs, but the details are unknown. Subsequently, the patient gradually developed limb numbness, accompanied by convulsions and unclear consciousness. She was sent to Fuxin Hospital in Liaoning Province 6 years ago for treatment and began taking the compound phenobarbital. Afterward, she did not experience any loss of consciousness attacks but still had numbness and convulsions in her limbs. For further diagnosis and treatment and of epilepsy, she came to the Tiantan Hospital affiliated with Capital Medical University 4 months ago. During imaging examinations, she was found to have a tumor in the cerebral cistern.

**Figure 1:**
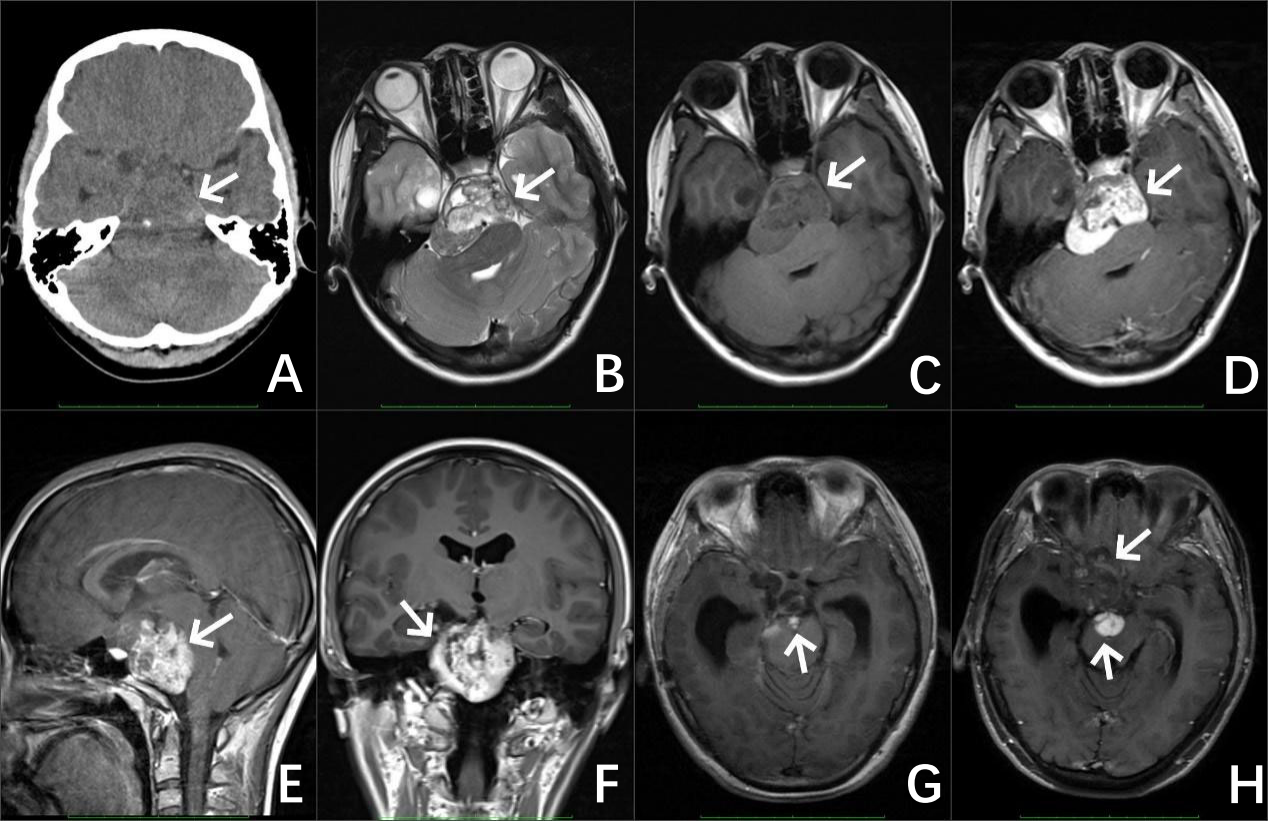
Preoperative image of the patient, CT of the brain **(A)** revealed an irregular jumble density mass with a well-defined boundary and a speckled calcification in the sellar region (as shown by the white arrow). MRI results of the brain **(B–F)** revealed irregular mixed signals and a markedly enhanced mass in the suprasellar cistern, ambient cistern, and interpeduncular cistern. Image of the patient after the operation **(G)** MRI of the brain revealed a few abnormal enhanced shadows at the operative area. Image of the patient 5 years after the operation **(H)** MRI of the brain revealed a significant increase in the area of abnormal enhancement at the surgical area.

CT of the brain showed an oblong slightly low-density shadow with a clear boundary that could be seen in the saddle area, measuring 42 mm × 49 mm × 47 mm, with a speckled calcification. The adjacent brain stem was compressed and shifted backward, and the bone in the clivus region was compressed and thinned. Magnetic resonance imaging (MRI) of the brain revealed irregular mixed-signal mass shadows in the suprasellar cistern, ambient cistern, and interpeduncular cistern, measuring 62 mm × 53 mm × 50 mm, with relatively clear boundaries and uneven enhancement. The brain stem was compressed and deformed, the optic chiasm and bilateral cavernous sinuses were unclear, and the supratentorial ventricles were dilated (**Figure 1**). An immature cholesteatoma was diagnosed based on the image. Subsequently, she underwent a right temporal craniotomy with an anterior inferior temporal petrosal approach for resection of the majority of the slope lesions.

**Figure 2:**
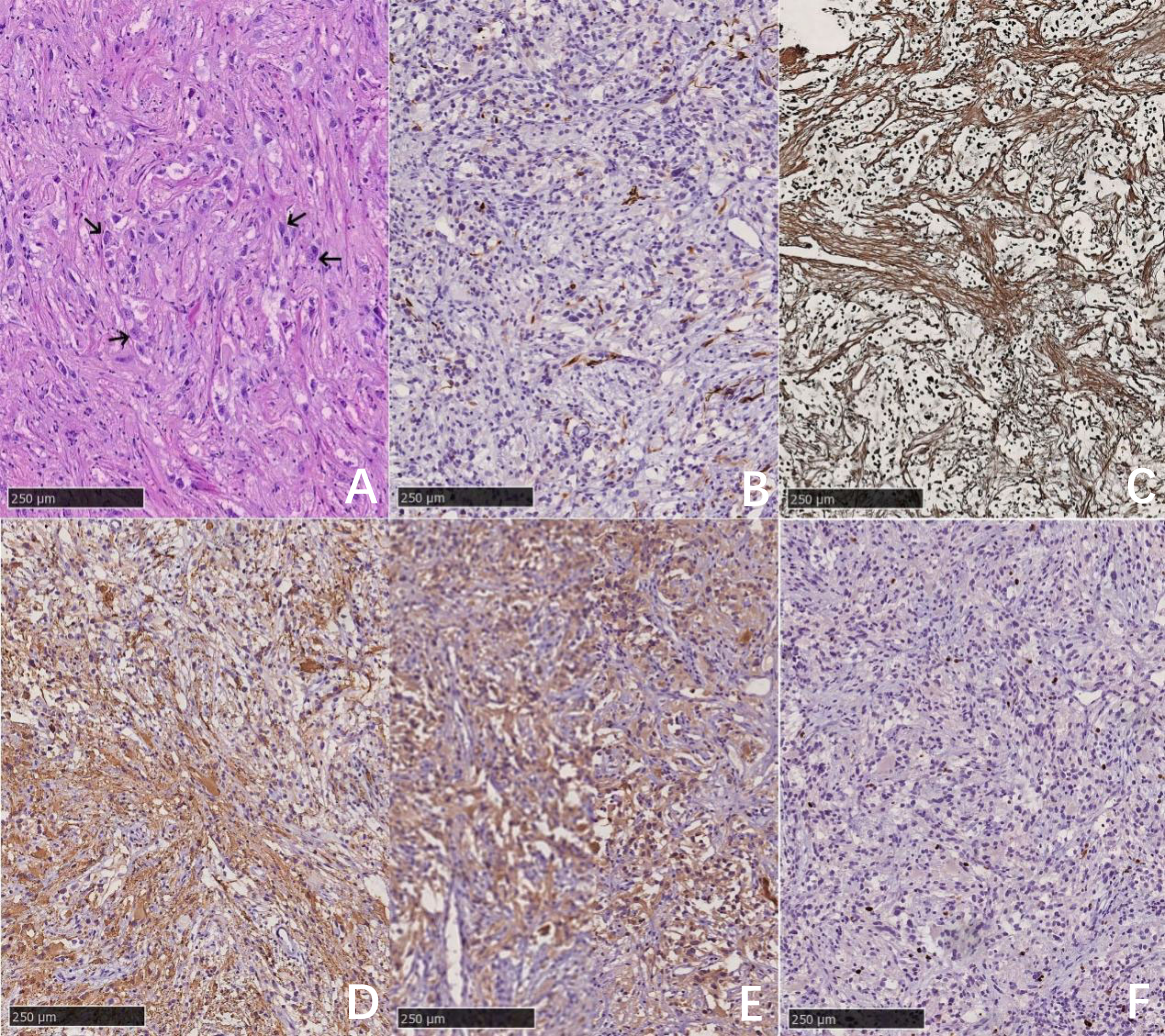
H&E stain. the tumor was mainly composed of fibroblast-like spindle cells and polymorphic astrocytes with radial-wheel, bundle, and vortex arrangement. The distribution of malformed neurons can be seen (as shown by the black arrow) **(A)**. The pathological diagnosis was desmoplastic infantile ganglioglioma (DIG). Immunohistochechemical staining: the tumor was positive for reticular fiber dyeing **(C)**, Syn **(D)**, and BRAFV600E **(E)**, and scattered positive for glial fibrillary acidic protein (GFAP) **(B)**, and Ki-67 was low (approximately 2%–5%) **(F)**.

During the operation, the tumor was found to be light yellow with clear boundaries and a rich blood supply. It wrapped around the basilar artery and adhered closely to the brainstem at the hard points, growing upward from the slope to the saddle area, and squeezing the pituitary stalk. Meanwhile, it adhered to the arterio-ocular nerve pushed down the brain stem, and reached below the facial nerve. A large part of the tumor was removed, measuring 45 mm × 40 mm × 35 mm, Histopathological analysis revealed a tumor composed of fibroblast-like spindle cells and polymorphic astrocytes with radial-wheel, bundle, and vortex arrangement. The distribution of malformed neurons can be seen. Immunohistochemical staining revealed that the tumor was positive for reticular fiber staining, BRAFV600E, Syn, Olig-2, and CD34 and scattered positive for glial fibrillary acidic protein (GFAP) and neuron-specific nuclear protein (NeuN), and Ki-67 was low (approximately 2%–5%). The pathology confirmed the diagnosis of DIG, classified as WHO Grade 1 (**Figure 2**). The patient did not receive any adjuvant therapy after surgery.

An MRI of the brain 3 months later revealed some abnormal signals in the surgical area, which were considered tumor residue. An MRI of the brain 2 months after revealed that the amount of abnormal enhancement in the surgical area was approximately the same as before, and a follow-up review was continued. The patient returned to the doctor 5 years later with frequent headaches, and a re-examination showed that the residual extent of the operative area was significantly larger than before and involved the right frontal and temporal lobes, considering the progression of the residual part of the tumor.

## 3 Discussion

DIG was first reported by Vandenberg et al. in 1987 (4). It shares similar clinical and neuroimaging features with desmoplastic infantile astrocytoma (DIA), including a favorable prognosis, but DIA lacks ganglion cells (5). DIG and DIA are currently classified as grade 1 neuronal and mixed neuronal–glial tumors by the WHO (2). DIG is a rare intracranial tumor in infants, usually occurring in infants and young children under 2 years of age, with a median age of onset of approximately 5–6 months (6). In recent years, there have also been a few reports of its occurrence in older children and adults (7). The clinical manifestations of DIG are non-specific, with the most common symptom being an enlarged head, followed by epilepsy, vomiting, headache, and so on (8). This tumor usually has a good prognosis, and most patients can be cured by surgery, but some have malignant features, such as multiple intracranial lesions (1, 9), postoperative progression or recurrence (3, 9, 10), meningeal diffusion, and metastasis (11).

The typical imaging manifestation of DIG is a cystic solid mass, mainly composed of cystic lesions, and calcification is rare. The cystic part is relatively large in volume and deep in position, while the solid part is close to the meninges and shallow in position, connected to the meninges by a wide base. In some cases, the adjacent skull bones may be compressed and thinned (8). Due to the presence of fibrous matrix components in the solid part of the tumor, both T1-weighted (T1W) images and T2-weighted (T2W) images show low signal intensity. The enhanced MRI scan shows significant enhancement in the solid part of the tumor, while there is no enhancement in the cystic part (12). The superficial and solid location of the lesion close to the meninges is considered a characteristic imaging manifestation of DIG (12). However, the case reported in this article differs from the majority of previous literature reports regarding the age, location, and imaging characteristics of DIG. In our study, we presented a 16-year-old girl with DIG who underwent progression 5 years after subtotal resection. The tumor was located in the suprasellar cistern, ambient cistern, and interpeduncular cistern and was mainly composed of solid components, accompanied by calcification. An enhanced MRI scan showed significant enhancement in the solid part of the tumor. Because diffusion-weighted imaging (DWI) was not performed at the time of imaging, and because the remaining imaging findings were similar to atypical cholesteatoma, this case resulted in a misdiagnosis.

The differential diagnosis for this tumor may include pleomorphic xanthoastrocytoma, ganglioglioma, pilocytic astrocytoma, and ependymoma. Pleomorphic xanthoastrocytoma is an astrocytoma with a relatively good prognosis that occurs at an older age, most often in children and young adults. The tumor is usually located on the surface of the cerebral hemisphere and involves the meninges. Typical histological features of the tumor include pleomorphic, lipid-containing cells, expressing GFAP and often surrounding reticular fibers and eosinophilic granulocytes, but lacking fibroproliferative features. Most patients have a long history of epilepsy (13). Ganglioglioma is mainly seen in children and adolescents, and most cases occur before the age of 30. The main clinical symptom is epilepsy. Tumors can occur in any part of the brain and spinal cord, not limited to superficial sites. DIG is generated by immature neuroepithelial cells and significantly dense connective tissue, whereas ganglioglioma lacks these structures (14). Pilocytic astrocytoma is a rare and slow-growing glioma that typically occurs in children and young adults (15). The cerebellum is the most common site of occurrence (16). The majority of the tumors presented as cystic masses with mural nodules, which were significantly enhanced after contrast agent injection (17). Ependymomas are common in children and young adults. Approximately 75% are located in the spinal canal, typically presenting as a solid heterogeneous mass with cysts, hemorrhage, and punctate calcifications. The tumor is closely associated with the fourth ventricle, and its characteristic feature is a plastic-like extension through the lateral recesses of the fourth ventricle into the cerebellopontine cistern (18, 19).

Total surgical removal is sufficient for the treatment of these tumors, and no chemotherapy or radiotherapy is indicated if complete resection is achieved, with a survival rate of 8 to 20 years, with approximately 10% mortality in recurrent cases (6). Therefore, the goal should be to achieve complete surgical resection as much as possible. However, some tumors cannot be completely resected due to their deep location, and the clinical effect of partial resections is poor. It has been reported that deep tumor location is a factor of increased mortality and an independent predictor of reduced time to tumor recurrence (20). As we reported in this case, due to the deep location of the tumor, the patient only underwent a partial resection of the tumor, and the postoperative pathological result was DIG. After 3 months of postoperative follow-up, the patient was found to have a small amount of residual tumor. She was found 2 years later to have no significant change in the residual tumor compared to before, and there was no active follow-up afterward. It was not until 5 years after surgery, when clinical symptoms appeared, that tumor progression was discovered. This further validates the notion that DIG has malignant biological characteristics and underscores the importance of careful clinical monitoring during follow-up. Therefore, close postoperative follow-up is necessary for patients with incomplete resection of deep tumors. For patients with residual tumors during follow-up, a second surgical resection and adjuvant chemotherapy and/or radiotherapy are required. At present, there is no consensus as to whether chemotherapy or radiotherapy should be used, as it is well known that chemotherapy has long-term destructive effects on the developing brain and that radiation causes mutations in DNA that can lead to the malignant transformation of normal tissues for many years later (21).

In summary, DIG is generally considered to be a benign tumor with a favorable prognosis. However, because of its diverse biological behavior, it is characterized by recurrence, progression, metastasis, and malignant transformation. Therefore, it is necessary for postoperative patients to undergo close follow-up, especially for those with deep tumors and subtotal resection.

## Data Availability

The original contributions presented in the study are included in the article/supplementary material. Further inquiries can be directed to the corresponding author.
